# EpCAM is a putative stem marker in retinoblastoma and an effective target for T-cell-mediated immunotherapy

**Published:** 2012-02-01

**Authors:** Moutushy Mitra, Mallikarjuna Kandalam, Anju Harilal, Rama Shenkar Verma, Uma Maheswari Krishnan, Sethuraman Swaminathan, Subramanian Krishnakumar

**Affiliations:** 1 L&T Department of Ocular Pathology, Vision Research Foundation, Tamil Nadu, India; 2 Department of Biotechnology, Indian Institute of Technology (IIT), Madras, India; 3 CeNTAB, SASTRA University, Tanjore, India

## Abstract

**Purpose:**

The molecular markers cluster of differentiation (CD)24, CD44, adenosine tri-phosphate (ATP) binding cassette protein G2 (ABCG2), and epithelial cell adhesion molecule (EpCAM) are widely used, individually or in combination, to characterize some types of cancer stem cells. In this study we characterized the EpCAM^+^ retinoblastoma (RB) cells for their cancer stem-like properties in vitro. Additionally, we targeted RB tumor cells via redirecting T cells using bispecific EpCAM×CD3 antibody.

**Methods:**

Flow cytometry was used to study the co-expression of EpCAM with putative cancer stem cell markers, such as CD44, CD24, and ABCG2, in RB primary tumors. In vitro methyl thiazol tetrazolium (MTT) assay, invasion assay, and neurosphere formation assay were performed to characterize EpCAM^+^ cells for their cancer stem/progenitor cell-like properties. We assessed the in vitro efficacy of bispecific EpCAM×CD3 antibody on RB tumor cell proliferation and validated the results by evaluating effector cytokine production in the culture medium with the ELISA method.

**Results:**

EpCAM was co-expressed with all cancer stem cell markers (CD44, CD24, and ABCG2) in primary RB tumors. EpCAM^+^ cells showed significantly higher proliferative invasive potential and neurosphere formation in vitro compared to EpCAM^–^ Y79 cells. EpCAM^+^ cells showed higher β-catenin expression compared to EpCAMˉ cells. EpCAM×CD3 significantly retarded proliferation of RB primary tumor cells. EpCAM×CD3 effectively induced the secretion of effector cytokines, such as interferon (IFN)-γ, tumor necrosis factor (TNF)-α, interleukin (IL)-10, IL-2, and transforming growth factor (TGF)-β1, and also perforin levels by pre-activated lymphocytes.

**Conclusions:**

EpCAM might be a novel cancer stem cell marker in RB. EpCAM×CD3 antibody redirecting T cells to attack RB tumor cells may prove effective in RB management. Further preclinical studies are needed to confirm the initial findings of our study.

## Introduction

Retinoblastomas (RBs), although rare, are the most frequent primary eye malignancy diagnosed in children. Retinoblastomas commonly invade through the sclera or along the optic nerve or hematogenously via the choroid. Distant metastasis is uncommon after early surgery and chemotherapy, and the 5-year survival rate is approximately 90%. Without treatment, retinoblastomas are almost universally fatal.

Presence of cancer stem cells in solid tumors have been shown to have a bad prognosis associated with drug resistance [1]. The existence of cancer stem-like cells has been reported in various malignancies, such as acute leukemia [2-4] and neural [5], breast [6,7], and ovarian tumors [8]. Previously, we reported that ATP binding cassette protein G2 (ABCG2) and mini chromosome maintenance protein 2 (MCM2) cancer stem cell (CSC)-like markers are expressed in retinoblastoma tumors [9], and recent reports have demonstrated that stem cells expressing Oct4, Nanog, ALDH1, and low Hoechst 33342 cells are present in human RB cells [10,11]. Epithelial cell adhesion molecule (EpCAM), an epithelial cell adhesion molecule earlier identified as a marker for stem/progenitor cells of adult liver and oval cells [12,13], seems to present all the characteristics of CSCs. Earlier; our group showed that EpCAM is highly expressed in RB tumors with invasion compared to tumors without invasion [14]. Subsequently, we demonstrated that impairment of the EpCAM gene leads to a marked decrease in cell proliferation [15]. However, there is no evidence as to whether EpCAM protein contributes to cancer stemness of RB tumor cells. In the present study we characterized the EpCAM^+^ cells for their cancer stem cell properties in vitro and evaluated EpCAM co-expression with cancer stem cell-like markers, such as cluster determinant (CD)44, CD24, and ABCG2, in fresh primary retinoblastoma tumors.

Bispecific antibodies (bsAb) are artificial molecules with dual specificity to two different antigens. The most commonly used antigen for bsAb on lymphocytes is an invariant CD3 signaling complex, which induces polyclonal T-cell activation. Several bsAb and single-chain antibody constructs against EpCAM have been generated and tested as immunotherapeutic agents [16-21]. Host antitumor immunity has been considered to play a role in protection against the development of malignancy. However, host mononuclear cells may become dysfunctional when there is a lack of expression of tumor-associated antigens or various co-stimulatory or major histocompatibility complex molecules in the tumor cells. Earlier we demonstrated that aggressive RB primary tumors express low levels of human leukocyte antigen (HLA) class I and class II antigens, which could be an advantage for tumor cells to escape from T-cell- or natural killer (NK) cell-mediated attack [22]. In this context, a novel therapeutic strategy that uses a bispecific antibody that redirects T cells to attack tumor cells could be an attractive treatment modality in retinoblastoma management. The bispecific antibody that we used has been shown to efficiently induce tumor cell lysis in vitro and reduce malignant ascites production in advanced ovarian cancer patients [23,24]. In the present study, besides demonstrating EpCAM as a cancer stem cell marker, we investigated the antitumor efficacy of EpCAM×CD3 bispecific antibody in retinoblastoma primary tumors.

## Methods

### Tissue collection

All samples were collected with the approval of the institutional review board at Vision Research Foundation (VRF) and in accordance with the Declaration of Helsinki. The retinoblastoma tumor samples were collected from the eyes enucleated from the retinoblastoma patients. When a patient is advised for enucleation as part of therapy, the eyeball will be sent to Department of Ocular Pathology for diagnostic purpose. According to the institutional ethics committee policies, a general consent is taken from all the patients who undergo enucleation. The age range and gender of all the patient samples included in this study is mentioned in the Table 1.Whatever clinicopathological parameters are required for marker correlations have been mentioned in the Table 1. Fresh unfixed eyeballs of patients with diagnosed RB, without any prior treatment, were included in the study. The enucleated eyeballs were obtained in sterile condition. After the fresh tissue was placed in plain RPMI medium, singly suspended cells were analyzed by flow cytometry. The slides were reviewed by an experienced ocular pathologist (S.K.) specifically for features of differentiation; histologic risk factors, such as involvement of optic nerve, choroid, anterior segment; and other associated features. The clinical profiles of the patients were obtained from their medical records.

**Table 1:** Clinicophenotypical correlation of markers in eight cases of Rb.

**Tumor number**	**Age/Sex**	**Clinicopathological features**	**% of EpCAM^+^/CD44+**	**% of EpCAM^+^/CD24+**	**% of EpCAM^+^/ABCG2+**
1	5 mon/M	OD, unilateral, Poorly Differentiated, Diffuse choroidal invasion and pre laminar/laminar optic nerve invasion	0.83	0.65	1.6
2	4Y/M	OD, unilateral, poorly differentiated, focal RPE and focal choroidal invasion.	32.6±3.6	14.2±1.9	6.5±1.5
3	2Y/M	OS, Bilateral, undifferentiated, focal choroidal and pre laminar optic nerve invasion	42±4.6	10.2	2.5±2.1
4	7 1/2 mon/M	OD, unilateral, well differentiated, focal choroidal with pre and post laminar optic nerve invasion.	1.1	29.7±2.8	3.7
5	32 mon/M	OD, unilateral, poorly differentiated, No invasion	14.1±2.1	15±4.2	3.8±0.33
6	1Y/F	OD, unilateral, moderately differentiated, pre laminar optic nerve invasion.	6.3±0.67	6.7±1.2	2.7±0.75
7	6Y/M	OD, unilateral, focal differentiation, full thickness choroidal invasion	23.8±3.2	20.7±2	1.97±1.8
8	1Y/F	OD, unilateral, well differentiated, focal RPE and pre laminar optic nerve invasion	42.6±4.1	41.4±2.8	4.0±0.2

Abbreviations: M: Male; F: Female; mon: months; Y: years; OD: right eye; RPE: retinal pigment epithelium.

### Flow cytometry

Single cell suspensions were prepared from tissues by mechanical disruption using syringe coupled with an 18G needle. Cells (2×10^5^ in each reaction) were suspended in 50 ml of fuorescence-activated cell sorting (FACS) buffer (0.5% BSA (BSA) and 0.02% sodium azide in phosphate buffer solution (PBS); 137 mM NaCl, 2.7 mM KCl, 4.3 mM Na_2_HPO_4_, 1.47 mM KH_2_PO_4_; pH adjusted to 7.4; Sigma-Aldrich, Saint Louis, MO) and blocked with FcR blocking reagent (2 µl/2×10^5^ cells; Miltenyi Biotech, Gladbach, Germany) for 15 min at 4 °C. Initially cell viability was tested using propidium iodide (PI) viability staining solution (eBioscience, San Diego, CA). Viable cells were defined by their forward scatter cell/side scatter cell (FSC/SSC) profiles and in addition, their lack of PI. Cells were then incubated for 45 min with a primary antibody for EpCAM, CD24, CD44, and ABCG2. Isotype controls for the corresponding antibodies used were as follows: antigen presenting cell (APC)-conjugated mouse IgG2a κ-isotype control (50 µg/0.5 ml), photoelectric (PE)-conjugated rat IgG2b isotype control (0.2 mg/ml), fluorescein isothiocyanate (FITC)-conjugated mouse IgG1 κ-isotype control (0.2 mg/ml), FITC-conjugated antimouse IgG1 (0.5 mg/ml; eBioscience, San Diego, CA). The antibodies used were mouse FITC-conjugated antihuman EpCAM (B29.1; 0.1 mg/ml; Abcam, Cambridge, UK), mouse PE-conjugated antihuman CD44 (F10, 0.1 mg/ml; Abcam), mouse APC-conjugated antihuman CD24 (ML5, 25 µg/ml; Biolegend, San Diego, CA), and mouse FITC-conjugated antihuman ABCG2 (CD3, 25 µg/ml; Biolegend).

Cells were washed with FACS buffer after antibody incubation and were analyzed with flow cytometry (CellQuest software; BD Biosciences, San Jose, CA) on a customized system (BD-FACS Calibur; BD Biosciences).

### Elimination of dead cells from the analysis

**Figure 1:**
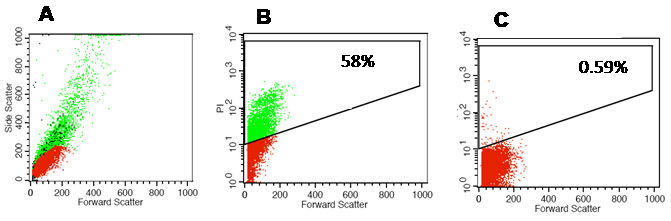
Elimination of dead cell population from the final analysis.**A**: Scatter plot showing most cells with small size and high granularity, which represent dead cells; hence the dead were eliminated from the final analysis. **B**: Plot showing 58% of the cells stained for PI were eliminated from the final analysis as the PI positive cells represent dead cell population. **C**: Gated scatter plot shows control cells without PI stain.

The RB tumor tissues included dead cells (ranging from 25.2%±2.3% to 75.1%±1.2%), which were characterized by their small size, high granularity, and positivity for PI dye. These were eliminated from the final analysis by appropriate gating (Figure 1A-C). Subsequently, the samples were evaluated for dead cells and marker expression in viable cells.

### Isolation of EpCAM^+^ cells from Y79 cell line

EpCAM^+^ cells were isolated from the Y79 cell line using CELLection Pan Mouse IgG kit (Invitrogen, Bangalore, India), according to the manufacturer’s instructions. Briefly, Dynabeads were incubated with EpCAM primary monoclonal antibody (Santa Cruz Biotechnology, Santa Cruz, CA) overnight, and the beads were then washed with 1% BSA in PBS (pH 7.4) to remove free unbound antibody. Approximately 3×10^6^ Y79 cells were then incubated with beads at 4 °C for 30 min on a tube rotator. After incubation, bead-bound cells were washed thrice with 1% BSA in PBS (pH 7.4) using a magnetic stand. The washed cells were resuspended in buffer containing RPMI 1640 and 1% fetal calf serum (FCS), 1 mM CaCl_2_, and 4 mM MgCl_2_ (pH 7.0–7.4). The cells were detached from the magnetic beads using releasing buffer (DNase 1) by incubating the cells at room temperature for 15 min. The released cells in the supernatant were collected carefully using magnetic beads for downstream applications.

### *EpCAM* siRNA transfection

After sorting out the EpCAM^+^ Y79 cells from the parental cell line, *EpCAM*-specific small interfering RNA (siRNA) treatment was performed as described in our previous report [15].

Briefly, for each well of cells, 200 nm of siRNA and 12 µl of Hi-perfect transfection reagent was mixed with 88 µl of serum free RPMI medium in a vial and incubated for 5–10 min at room temperature (RT) to allow the transfection complex formation. Then the complex was added drop-wise to the wells containing Y79 cells and the plate was gently swirled to ensure the uniform distribution of the transfection complex. The plates were incubated further for 6 h in a CO_2_ incubator at 37 °C. After 4 h of incubation, 400 µl of complete RPMI medium was added to the wells and incubated further for 24–72 h. Briefly, 1x10^5^ cells were plated in each well of six-well plates and allowed to grow for 24–36 h (until they were 40%–60% confluent). Small interefering RNA (siRNA) was then transfected into cells at a concentration of 200 nM using Hi-Perfect transfection reagent (Qiagen, Santa Clara, CA) and serum-free medium. After 4 h of incubation, serum-rich medium was added. Human *EpCAM* siRNA (Hs_TACSTD1_10; catalog number SI04343416; Forward strand: GGA ACU CAA UGC AUA ACU Att and the reverse strand: UAG UUA UGC AUU GAG UUC Cct) and scrambled siRNA (catalog number 1022563) were used in this study.

### In vitro cell proliferation assay

For the evaluation of the cell proliferation rate, cells were seeded on 96-well plates at a density of 1×10^4^ cells/well in complete RPMI medium, followed by the methyl thiazol tetrazolium assay (MTT assay; Sigma-Aldrich). The amount of MTT formazan produced was determined using a microplate reader and an absorbance of 560 nm (Beckman Coulter India Private Ltd, New Delhi, India)

### In vitro cell invasion analysis

We used a 24-well plate Transwell system with a polycarbonate filter membrane of 8-mm pore size (Corning, UK). The cell suspensions were seeded to the upper compartment of the Transwell chamber at a cell density of 1×10^5^ in 100 µl serum-free medium. After 24 h, the medium was removed and the filter membrane was fixed with 4% formalin for 1 h. The opposite surface of the filter membrane that faced the lower chamber was stained with methylene blue for 3 min, and the migrated cells were then visualized under an inverted microscope.

### Y79 cell neurospheres and differentiation assay

EpCAM^+^/EpCAMˉ/EpCAMˉsiRNA Y79 cells were plated in a 96-well dish at densities from 1,000 cells per well to 50 cells per well. The cells were plated at a low density (50 cells per well) to prevent the probability of the cells encountering one another in the well. After 5 days, neurospheres in the wells were counted and then dissociated into single-cell suspensions and replated. For the differentiation study, the sorted EpCAM^+^ (5,000 cells) and EpCAMˉ cells (5,000 cells) were cultured separately in complete RPMI medium for 2 weeks. After 14 days, the wells containing the EpCAM^+^ and EpCAMˉ cells were subjected to flow cytometry analysis to determine the percentage of EpCAM expression in both populations.

### Western blotting

Cells were lysed in RIPA lysis buffer for 15 min on ice. An aliquot (50 µg) of lysate was electrophoresed by 10% SDS-polyacrylamide gel and blotted onto a nitrocellulose membrane. Membranes were blocked in 5% fat-free milk and then incubated separately with 1:500 diluted mouse monoclonal primary antibody against EpCAM (C-10; Santa Cruz Biotechnology), EpCAM and β-catenin (E-5, 200 µg/ml; Santa Cruz Biotechnology) overnight at 4 °C. β-actin was used as the loading control (dilution 1:4,000, AC-15; Sigma Aldrich). After washing, membranes were incubated with horseradish peroxidase-conjugated antimouse IgG antibody (diluted to 1:2,000; Santa Cruz Biotechnology) for 1 h at room temperature. The bands were visualized using an enhanced chemiluminescence kit (Amersham, Pittsburgh, PA). Each experiment was performed in triplicate.

### Effector and target cell preparation

Primary RB cells isolated from RB-grossed eyeballs were grown in RPMI (Life Technologies, Karlsruhe, Germany) supplemented with 10% FCS (Life Technologies) at 37 °C in a 10% CO_2_-humidified incubator. Subconfluent cultures were harvested and checked for vitality by trypan blue exclusion. Only tumor cells with viability >95% were used for in vitro cytotoxicity assays. Peripheral blood mononuclear cells (PBMC) of healthy donors were used as effector cells for in vitro efficacy analysis of the bscEpCAM×CD3 antibody. PBMCs were prepared using vacutainers (BD Biosciences) from fresh blood collected from donors at the VRF laboratory. Purified PBMCs were cultured in RPMI/10% FCS and 25 mmol/l HEPES (Sigma-Aldrich) and activated with anti-CD3 antibody and recombinant IL-2. The relative numbers of different lymphocyte subpopulations (CD3-, CD4-, and CD8-positive lymphocytes) were determined by FACS analysis using specific monoclonal FITC-conjugated antibodies as described in the flow cytometry section.

### Activation of cytotoxic lymphocytes

To obtain activated PBMCs, density gradient purified cells were cultured at a concentration of 2×10^6^ cells/ml in medium containing OKT3 (5 µg/ml) and recombinant human IL-2 (20 units/ml; eBiosciences, San Diego, CA). After 4 days, cells were washed twice to remove remaining antibodies and cultured overnight in medium alone. On day 5, a cytotoxicity assay was performed.

### In vitro cytotoxicity assay

Bioactivity of bscEpCAM×CD3 was analyzed by a FACS-based in vitro cytotoxicity assay using RB tumor cells as target cells and human PBMCs (CD3-enriched) as effector cells. Target cells were washed twice with PBS and labeled with 1 μM of CM-DiI reagent RPMI medium at 37 °C for 5 min and then for additional 15 min at 4 °C. Labeled target cells were washed twice with RPMI/10% FCS and mixed with freshly isolated effector cells at an effector-to-target ratio of 10:1 or 5:1. Target (2×10^5^) and effector cells (1×10^6^ to 2×10^6^) in a volume of 50 µl RPMI/10% FCS were added per well in a 96-well round-bottomed plate. Serial dilutions of bscEpCAM×CD3 (fivefold or fourfold) were prepared in RPMI/10% FCS starting with a concentration of 1 pg/ml. Fifty microliters of the different bscEpCAM×CD3 solutions were added in triplicate to the corresponding wells.

Individual cytotoxicity mixtures were incubated for 0, 2, 8, 16, and 24 h at 37 °C/5% CO_2_. PI was added to a final concentration of 1 µg/ml per well, and plates were incubated for 10 min at room temperature. The number of CM-DiI and PI-positive target cells was determined by FACS. Cytotoxicity was measured as the ratio of PI-positive cells (dead cells) over total number of target cells (CM-DiI-positive) according to the formula cytotoxicity (%)=[(PI-positive cells/CM-DiI-positive cells) × 100]. Dose–response killing curves were analyzed, and the bscEp-CAM×CD3 concentration that induced half maximal killing (EC_50_ value) was calculated.

### Enzyme-linked immunosorbent assay

Target (2×10^5^) and effector cells (1×10^6^ to 2×10^6^) were incubated with EpCAM×CD3 or control EpCAM/CD3 antibodies (each at 1 µg/ml) for 72 h, and supernatants were harvested and kept frozen −80 °C until used. The production of tumoricidal cytokines, such as interferon-γ (IFN-γ), tumor necrosis factor-α (TNF-α), interleukin-2 (IL-2), IL-10, transforming growth factor-β1 (TGF-β1), and perforin by effector cells was measured by the ELISA method. Briefly, pre-coated ready-set-go ELISA plates were used to measure the cytokines. The supernatants were added to the wells in triplicates along with standards provided in separate wells and incubated at room temperature for 2 h followed by the incubation with detection antibody for 1 h at RT. After incubation, the solution was aspirated from the wells and incubated with Avidin-HRP for 30 min at RT. The plate was washed with wash buffer and incubated with TMB substrate solution for 15 min at room temperature. The plate was read at 450 nm. Color was developed with 3, 3′,5,5′ tetramethylbenzidine stabilized substrate (eBiosciences), stopped by adding 1 N H_2_SO_4_; OD at 450 nm was analyzed using an ELISA plate reader.

### Statistical analysis

All the experiments were performed in triplicate and the results presented as mean±SD. Statistical analysis was performed using the Student *t* test wherever appropriate. A p value <0.05 was considered to be statistically significant.

## Results

### Clinical profile

The clinical features of the eight cases are summarized in Table 1. The mean age of patients included in the study was 29.5±21.8 months (range 5 to 72 months) comprising six boys and two girls. All the tumors were sporadic cases, with right eye involvement in seven cases and left eye involvement in one case.

### Histologic examination

A summary of clinical and histologic findings is provided in Table 1. There were seven tumors associated with mild to severe invasion risk factors and one tumor without invasion. Among tumors with invasion, there were two tumors with diffuse choroidal invasion, two tumors with post-laminar optic nerve invasion, three tumors with focal choroidal invasion, and four tumors with pre-laminar optic nerve invasion. There were four tumors with poor differentiation, two tumors with moderate differentiation, and two tumors that were well differentiated.

### Phenotypic analysis of tumor cells

**Figure 2:**
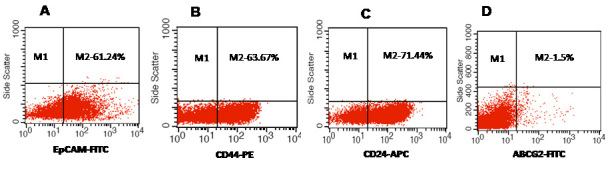
Percentage positivity of putative cancer stem cell markers in primary retinoblastoma (RB) tumors. **A**: Representative scatter plot shows 61.24% of epithelial cell adhesion molecule (EpCAM) positive cells in the RB tumor. **B**: Representative scatter plot shows 63.67% of cluster determinant (CD)44 positive cells in the RB tumors. **C**: Representative scatter plot shows 63.67% of CD24 positive cells in the RB tumors. **D**: Representative scatter plot shows 1.5% of ATP binding cassette protein G2 (ABCG2) positive cells in the RB tumors. EpCAM-FITC represents EpCAM^+^ cells identified using fluorescein isothiocyanate (FITC) labeled antibody. CD44-PE represents CD44 positive cells identified using phycoerythrin labeled antibody. CD24-APC represents CD24 positive cells identified using allophycocyanin labeled antibody. ABCG2-FITC represents ABCG2 positive cells identified using FITC labeled antibody.

**Figure 3:**
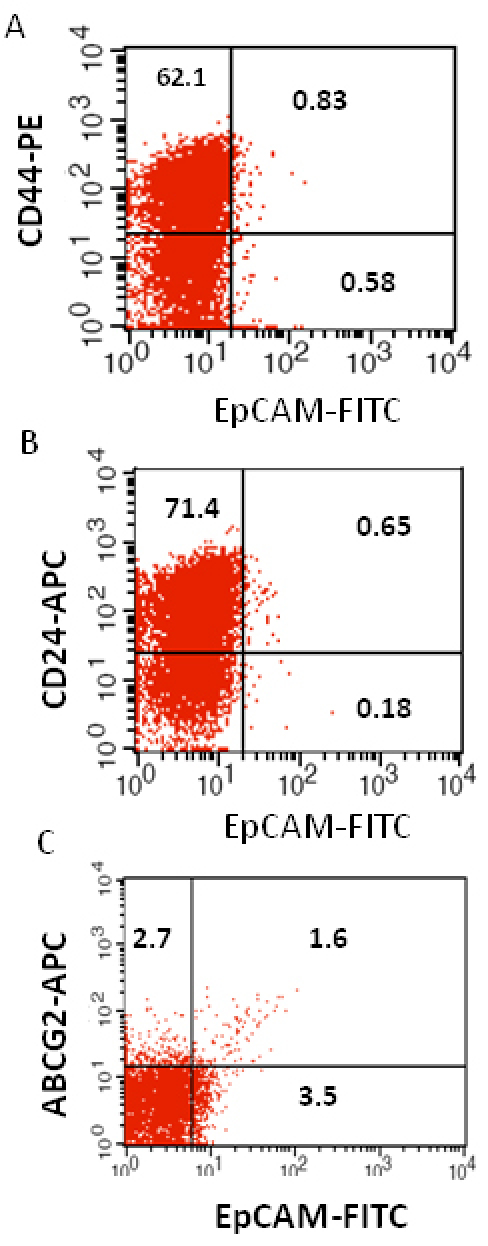
Investigation of the co-expression of epithelial cell adhesion molecule (EpCAM) with cluster determinant (CD)44, CD24 and ATP binding cassette protein G2 (ABCG2) markers in retinoblastoma (RB) tumor 1. **A**: Scatter plot shows the co-expression of EpCAM with CD44 **B**: Scatter plot shows the co-expression of EpCAM with CD24. **C**: Scatter plot shows the co-expression of EpCAM with ABCG2. EpCAM-fluorescein isothiocyanate (FITC) represents EpCAM^+^ cells identified using FITC labeled antibody. CD44-PE represents CD44 positive cells identified using phycoerythrin labeled antibody. CD24-APC represents CD24 positive cells identified using allophycocyanin labeled antibody. ABCG2-APC represents ABCG2 positive cells identified using allophycocyanin labeled antibody.

**Figure 4:**
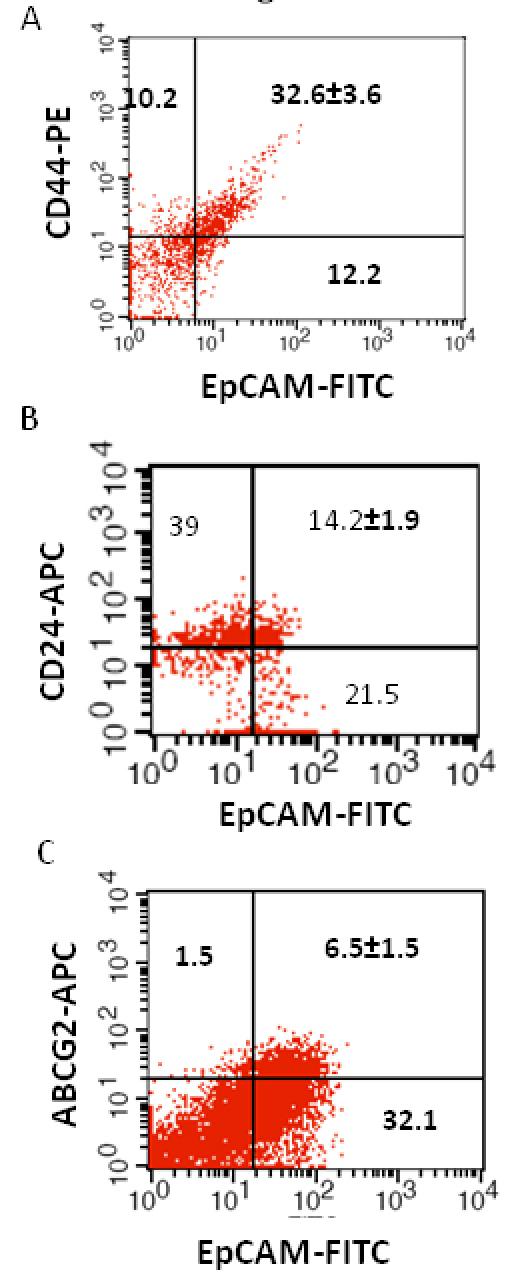
Investigation of the co-expression of epithelial cell adhesion molecule (EpCAM) with cluster determinant (CD)44, CD24 and ATP binding cassette protein G2 (ABCG2) markers in retinoblastoma (RB) tumor 2. **A**: Scatter plot shows the co-expression of EpCAM with CD44. **B**: Scatter plot shows the co-expression of EpCAM with CD24. **C**: Scatter plot shows the co-expression of EpCAM with ABCG2. EpCAM-fluorescein isothiocyanate (FITC) represents EpCAM^+^ cells identified using FITC labeled antibody. CD44-PE represents CD44 positive cells identified using phycoerythrin labeled antibody. CD24-APC represents CD24 positive cells identified using allophycocyanin labeled antibody. ABCG2-APC represents ABCG2 positive cells identified using allophycocyanin labeled antibody.

**Figure 5:**
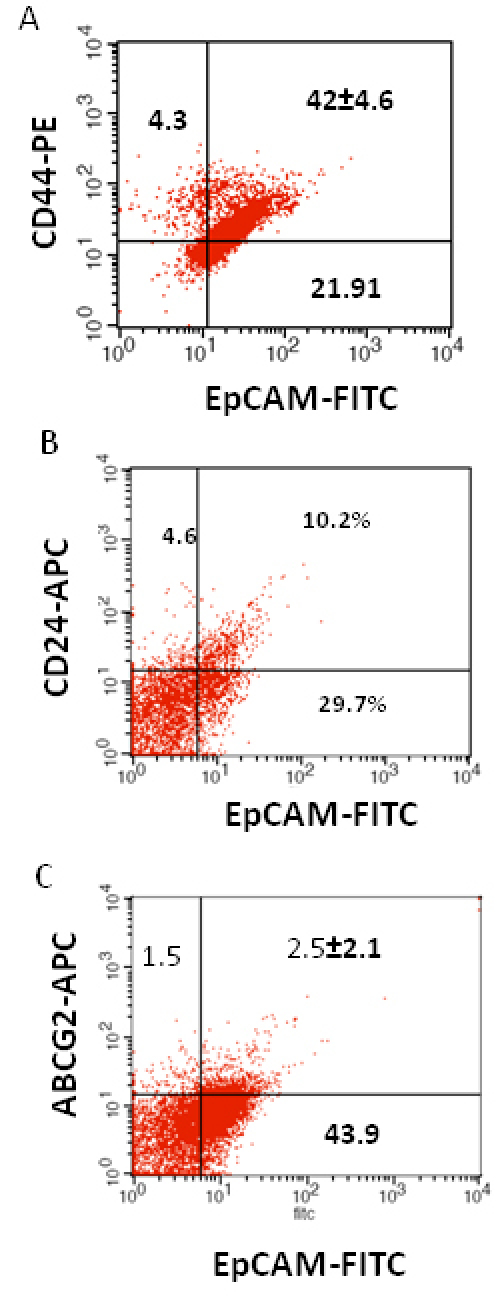
Investigation of the co-expression of epithelial cell adhesion molecule (EpCAM) with cluster determinant (CD)44, CD24 and ATP binding cassette protein G2 (ABCG2) markers in retinoblastoma (RB) tumor 3. **A**: Scatter plot shows the co-expression of EpCAM with Cluster determinant-44 (CD44). **B**: Scatter plot shows the co-expression of EpCAM with CD24. **C**: Scatter plot shows the co-expression of EpCAM with ABCG2. EpCAM-fluorescein isothiocyanate (FITC) represents EpCAM positive cells identified using FITC labeled antibody. CD44-PE represents CD44 positive cells identified using phycoerythrin labeled antibody. CD24-APC represents CD24 positive cells identified using allophycocyanin labeled antibody. ABCG2-APC represents ABCG2 positive cells identified using allophycocyanin labeled antibody.

**Figure 6:**
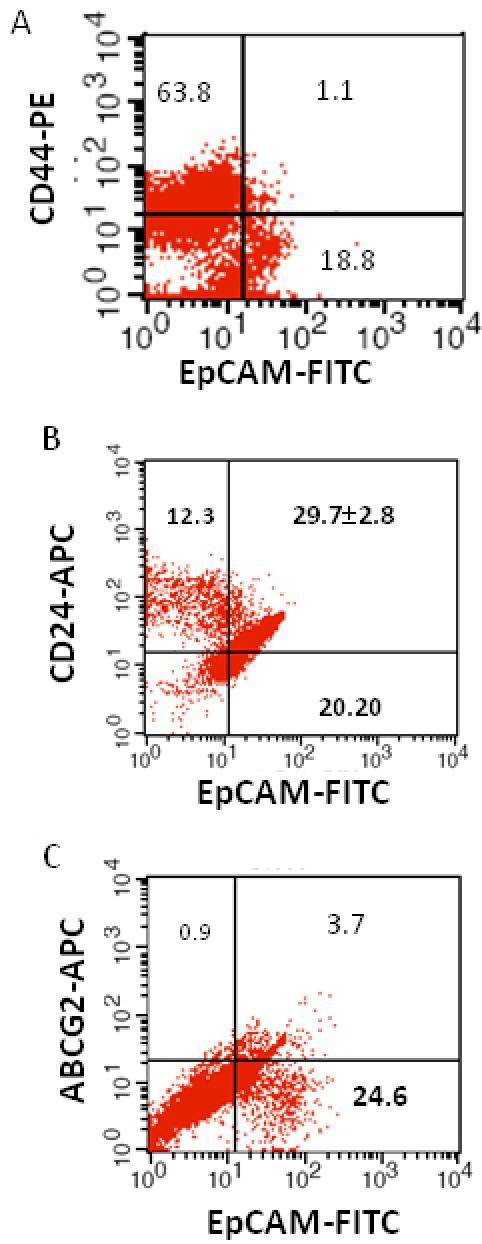
Investigation of the co-expression of epithelial cell adhesion molecule (EpCAM) with cluster determinant (CD)44, CD24 and ATP binding cassette protein G2 (ABCG2) markers in retinoblastoma (RB) tumor 4. **A**: Scatter plot shows the co-expression of EpCAM with CD44. **B**: Scatter plot shows the co-expression of EpCAM with CD24. **C**: Scatter plot shows the co-expression of EpCAM with ABCG2. EpCAM-fluorescein isothiocyanate (FITC) represents EpCAM positive cells identified using FITC labeled antibody. CD44-PE represents CD44 positive cells identified using phycoerythrin labeled antibody. CD24-APC represents CD24 positive cells identified using allophycocyanin labeled antibody. ABCG2-APC represents ABCG2 positive cells identified using allophycocyanin labeled antibody..

**Figure 7:**
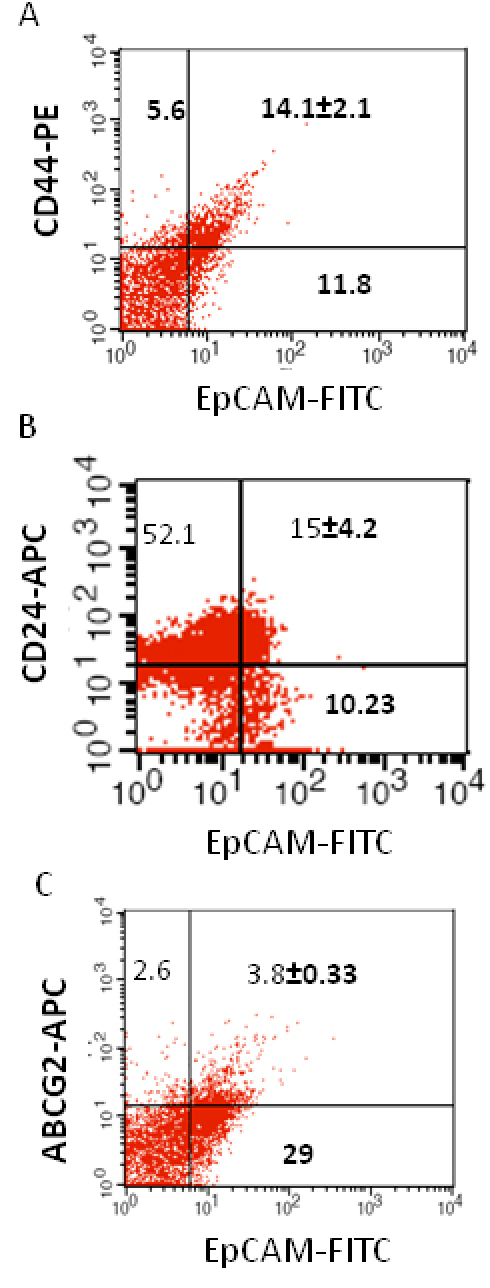
Investigation of the co-expression of epithelial cell adhesion molecule (EpCAM) with cluster determinant (CD)44, CD24 and ATP binding cassette protein G2 (ABCG2) markers in retinoblastoma (RB) tumor 5. **A**: Scatter plot shows the co-expression of EpCAM with CD44. **B**: Scatter plot shows the co-expression of EpCAM with CD24. **C**: Scatter plot shows the co-expression of EpCAM with ABCG2. EpCAM-fluorescein isothiocyanate (FITC) represents EpCAM positive cells identified using FITC labeled antibody. CD44-PE represents CD44 positive cells identified using phycoerythrin labeled antibody. CD24-APC represents CD24 positive cells identified using allophycocyanin labeled antibody. ABCG2-APC represents ABCG2 positive cells identified using allophycocyanin labeled antibody.

**Figure 8:**
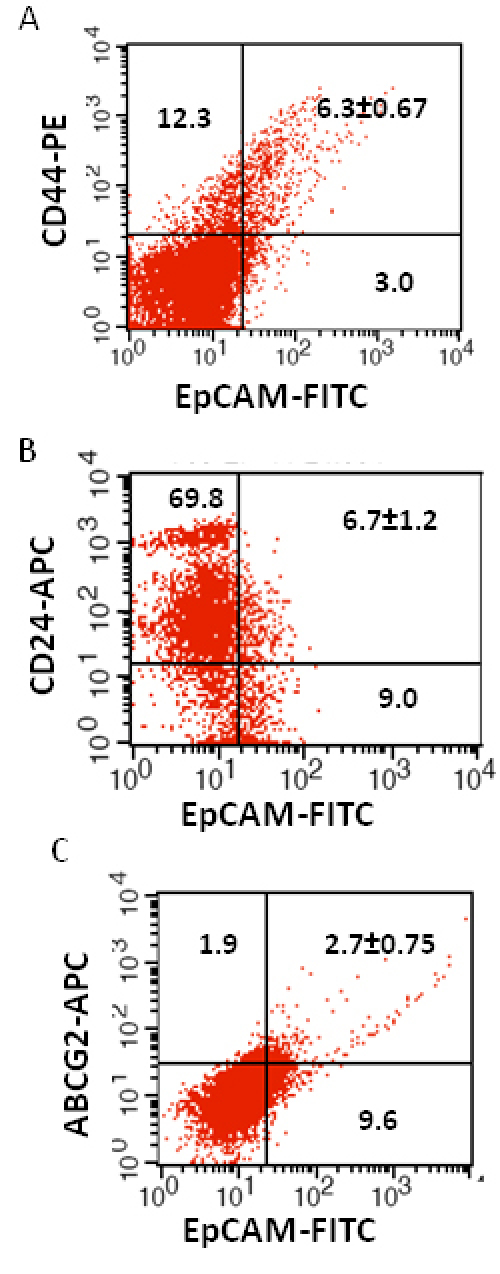
Investigation of the co-expression of epithelial cell adhesion molecule (EpCAM) with cluster determinant (CD)44, CD24 and ATP binding cassette protein G2 (ABCG2) markers in retinoblastoma (RB) tumor 6. **A**: Scatter plot shows the co-expression of EpCAM with CD44. **B**: Scatter plot shows the co-expression of EpCAM with CD24. **C**: Scatter plot shows the co-expression of EpCAM with ABCG2. EpCAM-fluorescein isothiocyanate (FITC) represents EpCAM positive cells identified using FITC labeled antibody. CD44-PE represents CD44 positive cells identified using phycoerythrin labeled antibody. CD24-APC represents CD24 positive cells identified using allophycocyanin labeled antibody. ABCG2-APC represents ABCG2 positive cells identified using allophycocyanin labeled antibody.

**Figure 9:**
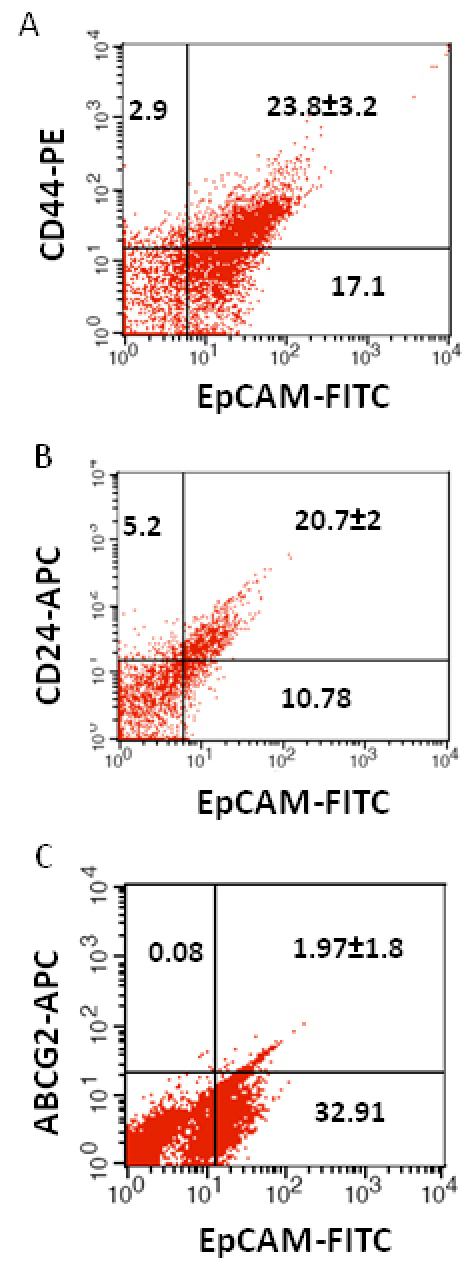
Investigation of the co-expression of epithelial cell adhesion molecule (EpCAM) with cluster determinant (CD)44, CD24 and ATP binding cassette protein G2 (ABCG2) markers in retinoblastoma (RB) tumor 7. **A**: Scatter plot shows the co-expression of EpCAM with CD44. **B**: Scatter plot shows the co-expression of EpCAM with CD24. **C**: Scatter plot shows the co-expression of EpCAM with ABCG2. EpCAM-fluorescein isothiocyanate (FITC) represents EpCAM positive cells identified using FITC labeled antibody. CD44-PE represents CD44 positive cells identified using phycoerythrin labeled antibody. CD24-APC represents CD24 positive cells identified using allophycocyanin labeled antibody. ABCG2-APC represents ABCG2 positive cells identified using allophycocyanin labeled antibody.

**Figure 10:**
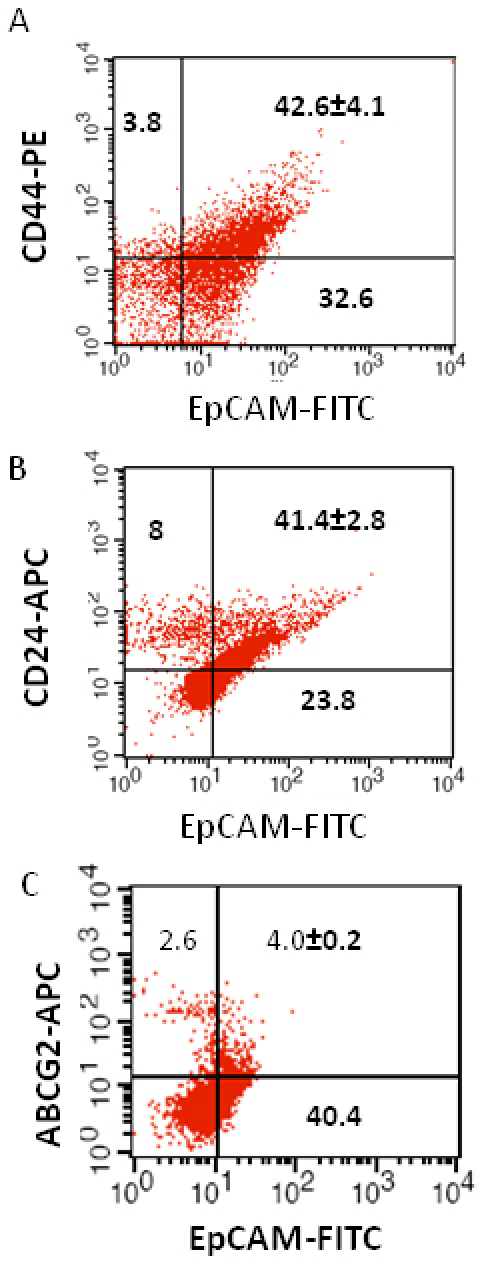
Investigation of the co-expression of epithelial cell adhesion molecule (EpCAM) with cluster determinant (CD)44, CD24 and ATP binding cassette protein G2 (ABCG2) markers in retinoblastoma (RB) tumor 8. **A**: Scatter plot shows the co-expression of EpCAM with CD44. **B**: Scatter plot shows the co-expression of EpCAM with CD24. **C**: Scatter plot shows the co-expression of EpCAM with ABCG2. EpCAM-fluorescein isothiocyanate (FITC) represents EpCAM^+^ cells identified using FITC labeled antibody. CD44-PE represents CD44 positive cells identified using phycoerythrin labeled antibody. CD24-APC represents CD24 positive cells identified using allophycocyanin labeled antibody. ABCG2-APC represents ABCG2 positive cells identified using allophycocyanin labeled antibody.

We investigated the expression of four markers that have previously been described for isolation of several types of human CSCs in human cancer cell lines: CD44, CD24, ABCG2, and EpCAM [25-32]. In this study CD44, CD24, ABCG2, and EpCAM could be detected by flow cytometry in all the RB patient tumor samples. The individual expression of CD44, CD24, ABCG2, and EpCAM in primary tumor samples ranged from 19% to 64%, 15% to 77%, 2% to 7%, 4% to 65%, respectively (Figure 2). The co-expression of EpCAM^+^CD44^+^, EpCAM^+^CD24^+^, and EpCAM^+^ABCG2^+^ ranged from 0.83% to 42.6%, 0.65% to 41.4%, and 1.6% to 6.5%, respectively, in the eight RB tumor samples (Figure 3, Figure 4, Figure 5, Figure 6, Figure 7, Figure 8, Figure 9, and Figure 10). The percentage of cancer cells expressing a single surface marker in individual tumors was significantly different. Except tumor No.1, all other tumors showed a significantly higher percentage of cells positive for the combination markers. The percentage of ABCG2 was less in all eight tumors, which is consistent with a previous study [31]. Although the expression of AGCB2 was less in tumors, we could still see that a small subpopulation of ABCG2-positive cells co-expressed EpCAM.

### Isolation and characterization of EpCAM^+^ cells in the Y79 cell line

**Figure 11:**
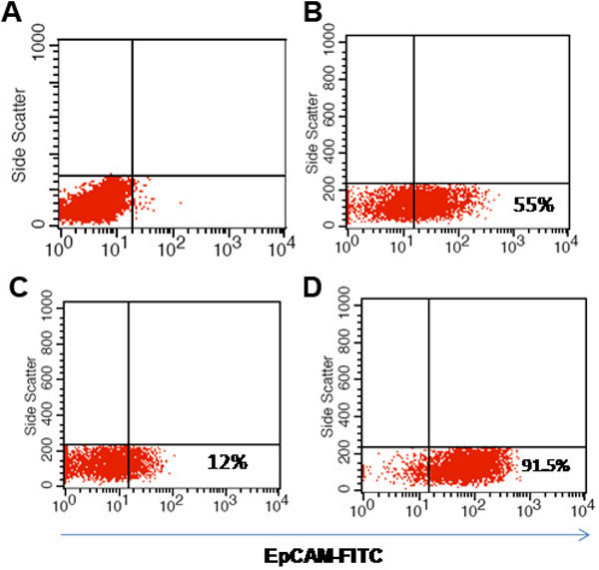
Determining the purity of epithelial cell adhesion molecule (EpCAM)^+^ cells in the magnetic bead isolation process.**A**: Scatter plots showing unstained cells for EpCAM as control. **B**: Scatter plot shows EpCAM expression in the native Y79 cells before subjecting to magnetic bead isolation assay. **C**: Scatter plot shows the EpCAM expression in the Y79 cells that were not bound to the magnetic beads.**D**: Scatter plot shows EpCAM expression in the Y79 cells sorted using EpCAM antibody coupled to magnetic beads.

Next, we demonstrated that EpCAM^+^ cells possess cancer stem cell-like properties. We successfully enriched EpCAM^+^ and EpCAMˉ populations from Y79 cells by using a magnetic cell sorting technique; this produced 90% purity in EpCAM^+^ cells and 90% purity in EpCAMˉ cells 1 day after sorting (Figure 11).

### Differentiation potential of EpCAM^+^ cells

**Figure 12:**
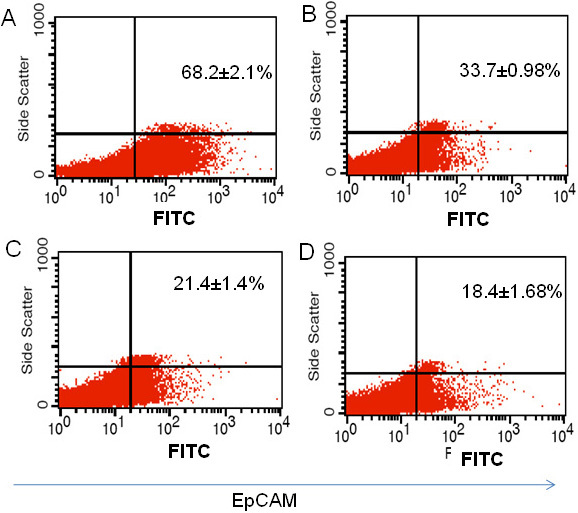
Demonstration of differentiation potential of epithelial cell adhesion molecule (EpCAM)^+^ cells. **A**: Scatter plot shows EpCAM expression in sorted EpCAM^+^ population at 1st day of culture. **B**: Scatter plot shows EpCAM expression in sorted EpCAM^+^ population at day 14th day of culture. **C**: Scatter plot shows EpCAM expression in unsorted EpCAM^-^ population at 1st day of culture. **D**: Scatter plot shows EpCAM expression in sorted EpCAM^+^ population at 14th day of culture.

In the differentiation assay the initially sorted EpCAM^+^ (81%) RB population ultimately decreased in time to 33.7% of the EpCAM^+^ fraction after 14 days of culture (Figure 12). However, a small percentage of EpCAM^+^ fractions remained stable in sorted EpCAMˉ RB cells even after 14 days of culture (Figure 12).

### Proliferation and invasion potential of EpCAM^+^ Y79 cells

**Figure 13:**
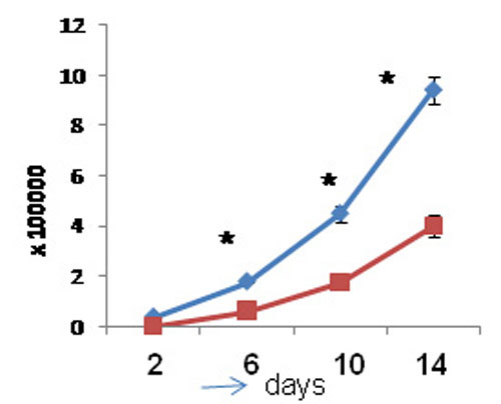
Comparison of cell proliferation kinetics between epithelial cell adhesion molecule (EpCAM)^+^ and EpCAM^-^ populations. The methyl thiazol tetrazolium (MTT) assay graph shows higher cell proliferation rate in EpCAM^+^ population compared to that of EpCAM^-^ population. The viability was determined in both populations at day 2, 6, 10 and 14. Statistically significant increased in cell viability of EpCAM^+^ population was observed at 6th, 10th and 14th day. Asterisk mark represent p value less than 0.05 as analyzed by *t*-test and the error bars represent standard error mean obtained from triplicate values.

**Figure 14:**
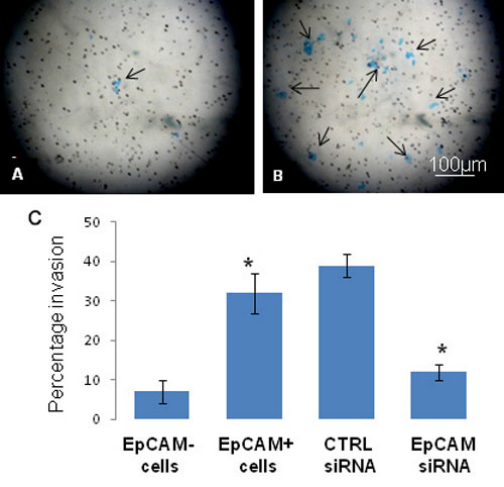
Comparison of invasion property between epithelial cell adhesion molecule (EpCAM)^+^ and EpCAM^-^ cells. **A**: Representative microscopy picture showing the invasion of EpCAM^–^ Y79 cells. **B**: Representative microscopy picture showing the invasion of EpCAM^+^ Y79 cells. **C**: Bar diagram showing increased number of EpCAM^+^ cells (bar 2) invading the matrigel compared to EpCAM^–^ cells (bar 1). Smilarly, bar 4 shows decrease in the invasion of EpCAM^+^ cells treated with EpCAM specific small interfering RNA when compared to small interfering RNA (siRNA) untreated EpCAM^+^ cells (bar 3). The error bars represent standard error mean of triplicate values. For statistical analysis, group 2 was compared with group 1 and group 4 was compared to group 3. Asterisk mark represent p value less than 0.05 which is statistically significant as analyzed by *t*-test.

Having isolated the EpCAM^+^ cells from Y79 cells, we next determined their cancer stem-like cell properties. EpCAM^+^ cells had a higher proliferation rate than EpCAMˉ cells, as assessed by the MTT assay (p<0.05, Figure 13) and were more invasive in in vitro matrigel Transwell invasion assays when compared to EpCAMˉ cells (p<0.05; Figure 14B). Similarly, when EpCAM^+^ cells were treated with EpCAM^–^ siRNA in vitro for 48 h, the invasive potential was decreased significantly (p<0.05; Figure 14B) compared to siRNA untransfected EpCAM^+^ cells.

### Neurosphere formation potential of EpCAM^+^ Y79 cells

**Figure 15:**
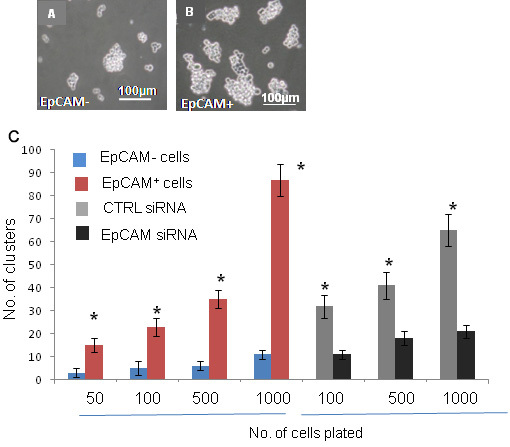
Comparison of neurosphere forming potential between epithelial cell adhesion molecule (EpCAM)^+^ and EpCAM^-^ cells. **A**: Phase contrast microscope image showing the formation of neurospheres by EpCAM^–^ cells in the cell culture plates. **B**: Phase contrast microscope image showing increased number of neurospheres by EpCAM^+^ cells in the cell culture plates. **C**: Bar diagram showing the number of colonies formed by EpCAM^+^ cells and EpCAM^-^ cells. Red bars indicate number of neurosphere clusters formed by EpCAM^+^ cells whereas blue bars indicate the number of neurosphere clusters formed by EpCAM^-^ cells. Asterisk mark over red bars indicate statistically significant (p<0.05) increase in number of neurospheres as analyzed by *t*-test. Black bars indicate decrease in number of neurospheres by EpCAM+ cells after siRNA treatment. Grey bars indicate number of neurospheres formed by small interfering RNA (siRNA) untreated EpCAM^+^ cells (control cells–CTRL). Asterisk mark over black bar indicates statistically significant (p<0.05) decrease in number of neurospheres as analyzed by *t*-test. The error bars represent standard error mean of triplicate values.

We examined EpCAM^+^/EpCAMˉ Y79 cells for their capacity to form neurospheres, an indicator of stem cell self-renewal [33]. The isolated EpCAM^+^ cells formed neurospheres efficiently, while EpCAM^–^ cells failed to do so (Figure 15A). After 5 days, neurospheres were counted and results presented, as shown in Figure 15B. Furthermore, neurospheres could be repeatedly passaged. It appears that EpCAM^+^ cells but not EpCAM^−^ cells have self-renewal capabilities with the ability to form colonies from a single cell. To confirm the findings, EpCAM^+^-sorted cells were treated with EpCAM^–^ siRNA in vitro for 48 h, and we observed that the number of neurospheres decreased significantly (p<0.05; Figure 15B) compared to siRNA untransfected EpCAM^+^ cells.

### Higher β-catenin expression in EpCAM^+^ cells

**Figure 16:**
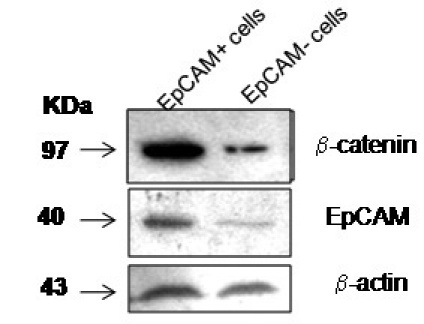
Western blotting analysis demonstrating the levels of epithelial cell adhesion molecule (EpCAM) and β-catenin levels in EpCAM^+^ and EpCAM^-^ cells. Panel 1 shows decreased levels of β-catenin in EpCAM^-^ cells compared to that of EpCAM^+^ cells. Panel 2 shows decreased EpCAM levels in EpCAM- cells compared to EpCAM^+^ cells. Panel 3 shows equal expression of β-actin (loading controls) in both EpCAM^+^ and EpCAM^-^ cells. kDa stands for kilo-dalton.

The isolated EpCAM^+^ cells showed increased β-catenin expression compared to EpCAMˉ cells (Figure 16), indicating that EpCAM might be involved in the β-catenin-mediated self-renewal ability of tumor cells.

### Effect of EpCAM×CD3 against human retinoblastoma cells

**Figure 17:**
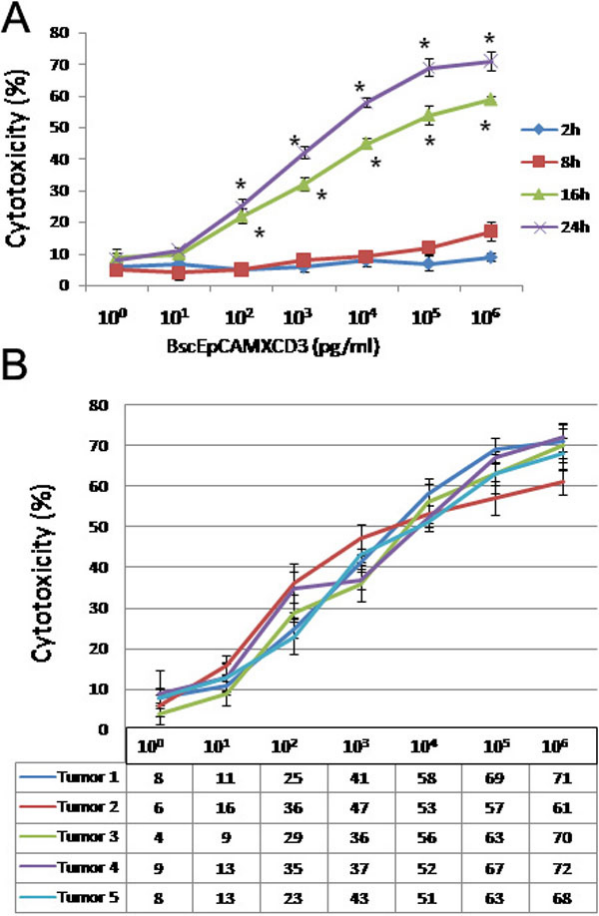
Varying concentrations of epithelial cell adhesion molecule (EpCAM)xcluster determinant (CD)3 antibody inducing cytotoxicity on retinoblastoma (RB) tumor cells. **A**: Graph shows the effect of increasing serial 10-fold concentrations of EpCAMxCD3 on the cytotoxicity of RB tumor cells at different incubation periods. The error bars represent standard error mean of triplicate values. Asterisk mark represent statistically significant (p<0.05; analyzed by Analysis of Variance method) increase in cytotoxicity of RB tumor cells at 16 h and 24 h incubation periods compared to that of 2 h and 8 h incubation periods. **B**: Effect of varying concentrations of EpCAMxCD3 on five RB tumors. The graph shows the cytotoxic effects of various concentrations of EpCAM×CD3 antibody on five RB primary tumors after 24 h of incubation. The error bars represent standard error mean of triplicate values. The table under the graph represents the percentage cytotoxicity of each RB tumor at particular concentration of EpCAMxCD3 antibody.

The in vitro efficacy of EpCAM×CD3 antibody was tested in a FACS-based cytotoxicity assay using CD3-positive pre-activated PBMCs of healthy donors as effector cells and EpCAM-positive human RB cells as target cells (tumor 1). There was a strong dose-dependent effect of EpCAM×CD3 on RB cells over a 24-h time period (Figure 17A). We did not observe significant tumor cell lysis after 2 h of incubation in the presence of EpCAM×CD3. However, tumor cell lysis occurred after 8 h of incubation but remained low with 17% lysis above background levels. Following 16 h of incubation, maximal specific lysis reached 59% and EC_50_ was reached at an EpCAM×CD3 concentration of 20 ng/ml. A 24-h incubation resulted in 71% specific lysis at 1 µg/ml EpCAM×CD3 with an EC_50_ value of 2 ng/ml.

To validate the results and test for reproducibility, we tested the efficacy of EpCAM×CD3 on four more tumor samples that were positive for EpCAM. Overall, we observed a mean tumor cell death of 68.4% out of the five tumors, ranging from 61% to 72% cell death (Figure 17B).

### Secretion of cytokines IFN-γ, TNF-α, and interleukins by pre-activated lymphocytes in the culture medium

**Figure 18:**
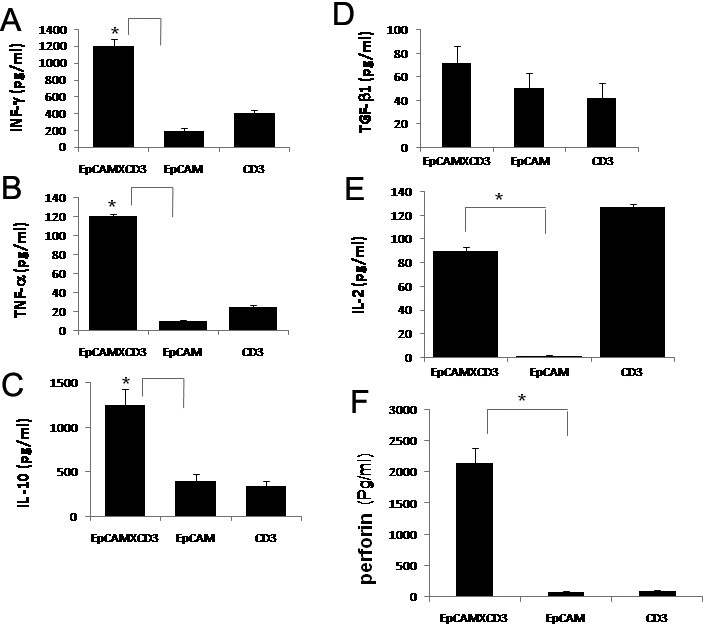
Production of cytokines by T-cells in the culture supernatant containing retinoblastoma (RB) tumor cells incubated with or without epithelial cell adhesion molecule (EpCAM)xcluster determinant (CD)3 antibody. **A**: Bar diagram shows statistically significant (*p<0.05; by *t*-test) increase in the levels of interferon-gamma (INF-γ) in the culture supernatant incubated with EpCAMxCD3 (Epithelial cell adhesion molecule X cluster determinant-3) antibody compared to that of EpCAM antibody. **B**: Bar diagram shows statistically significant (*p<0.05; by *t*-test) increase in the levels of tumor necrosis factor alpha (TNF-α) in the culture supernatant incubated with EpCAMxCD3 antibody compared to that of EpCAM antibody. **C**: Bar diagram shows statistically significant (*p<0.05; by *t*-test) increase in the levels of interleukin-10 (IL-10) in the culture supernatant incubated with EpCAMxCD3 antibody compared to that of EpCAM antibody. **D**: Bar diagram shows increase in the levels of transforming growth factor-β1 (TGF-β1) in the culture supernatant incubated with EpCAMxCD3 antibody compared to that of EpCAM antibody. **E**: Bar diagram shows statistically significant (*p<0.05; by *t*-test) increase in the levels of interleukin-2 (IL-2) in the culture supernatant incubated with EpCAMxCD3 antibody compared to that of EpCAM antibody. **F**: Bar diagram shows statistically significant (*p<0.05; by *t*-test) increase in the levels of perforin in the culture supernatant incubated with EpCAMxCD3 antibody compared to that of EpCAM antibody. Error bars in the entire bar diagrams represent standard error mean of triplicate values.

After 72 h of incubation, we observed increased levels of IFN-γ, TNF-α, IL-10, IL-2, and TGF-β1 in the supernatant of RB tumor cell and PBMC cultures incubated with 1 µg/ml of EpCAM×CD3 antibody compared to an incubation with CD3 or EpCAM monoclonal antibodies alone (Figure 18). There was also a significant increase in perforin levels with EpCAM×CD3 after 72 h of incubation compared with control antibodies (Figure 18E).

## Discussion

EpCAM has not been associated with any of the cancer stem cell markers in retinoblastoma. Using flow cytometry, we studied the individual expression of EpCAM, CD44, CD24 and ABCG2 and also studied the co-expression of EpCAM with other three putative cancer stem cell markers. All the markers expressed at different levels in 8 tumor samples. We observed that a subpopulation of tumor cells in all the samples showed dual expression of EpCAM with CD44, CD24 and ABCG2 individually (Figure 1). Thus, the present data clearly showed that EpCAM is co-expressed with three other cancer stem-like cell markers. Previous studies have shown CD44 to be the hallmark of cancer stem cells in leukemia, pancreatic, and breast cancers [34-39]. The expression of CD44 in primary RB tumors could be due to the presence of hyaluronic acid in the vitreous fluid [31].

EpCAM-positive cells isolated from the Y79 cell line demonstrated a self-renewal capability by forming a significantly higher number of neurospsheres in vitro and showing increased invasion potential in the matrigel in vitro. These results suggest that EpCAM expression might play a significant role in promoting the neurospheres formation and EpCAM may be associated with RB tumor invasion property . To a large extent, EpCAM^+^ cells turned to EpCAM^–^ cells, which indicate that EpCAM^+^ cells demonstrate a differentiation potential, a characteristic feature of cancer stem cells. Thus, it is clear that EpCAM^+^ Y79 cells behave like cancer stem cells. This was further evidenced by the presence of increased expression β-catenin, a critical player for maintaining embryonic stem cells [39], in the isolated EpCAM^+^ Y79 cells compared to EpCAM¯ cells. Increased β-catenin levels are produced in cells due to the activation of canonical Wingless and Int1 (Wnt) pathway activation [40]. The Wnt signaling pathway is implicated in the self-renewal and differentiation of stem cells [39]. A controversial study was published by Shoshana et al. [41] that reported the Wnt signaling pathway has tumor suppressor properties in the retinoblastoma cell line. However, a recent study by Amanda et al. [10] demonstrated that the GSK-3 β inhibitor lithium chloride activates Wnt signaling in retinoblastoma Y79 and WERI cell lines. While the ambiguity in these two different controversial data sets needs to be addressed, one possible reason for the difference is the differences in the cell lines used.

Our study shows for the first time that EpCAM^+^ RB cells behave like cancer stem cells. However, further in vivo studies need to be performed to confirm these findings. Therefore, targeting CSC pools together with conventional chemotherapies should be an essential treatment strategy to eradicate a tumor mass [42]. Both EpCAM and CD3 molecules are attractive targets for the generation of bsAb and bispecific single-chain antibody constructs for anticancer treatment [43-45].

We observed that pre-activated PBMC’s have effectively caused lysis of RB tumor cells in the presence of EpCAM×CD3. This effect was consistently noticed in all 5 tumors studied. The activity of EpCAM×CD3 was dose-dependent and increased over a 24-h time period. Our ELISA assays have demonstrated increase in effector cytokines production in the supernatants of culture containing EpCAM^+^ cells and pre-activated PBMC’s along with EpCAM×CD3. Taken together, our results are consistent with the study of Salnikov et al. [46] in that EpCAM×CD3 potently stimulates pre-activated lymphocytes to secrete effector cytokines in the presence of EpCAM expressing tumor cells. Secretion of TNF-α, IFN-γ, and chemokines by activated T cells may add to a therapeutic effect through intensive immune cell attraction and stimulation. High levels of IL-10 within the tumor microenvironment have been shown to favor tumor rejection by enhancing cytotoxicity of T lymphocytes [47]. On the other hand it has been shown that TGF-β has a tumor cell inhibitory function [48], and our results in the present study suggest that the bi-specific antibody mediated immunotherapy approach might help control RB tumor cell proliferation. A high proportion of cells in retinoblastoma express EpCAM, and tumors with optic nerve/choroidal invasion in particular show increased EpCAM expression [14]. Therefore, invasive retinoblastoma tumors are interesting tumors for target therapy using bispecific antibodies (EpCAM×CD3). In conclusion, we showed that EpCAM^+^ RB cells behave like cancer stem cells in vitro. EpCAM×CD3 possesses potent antitumor activity in vitro by inducing secretion of interleukins and cytokines by pre-activated lymphocytes in the presence of EpCAM-expressing RB tumor cells.
